# Short-Term Associations between PM_10_ and Respiratory Health Effects in Visby, Sweden

**DOI:** 10.3390/toxics10060333

**Published:** 2022-06-17

**Authors:** Andreas Tornevi, Henrik Olstrup, Bertil Forsberg

**Affiliations:** Section of Sustainable Health, Department of Public Health and Clinical Medicine, Faculty of Medicine, Umeå University, 901 87 Umeå, Sweden; andreas.tornevi@umu.se (A.T.); bertil.forsberg@umu.se (B.F.)

**Keywords:** PM_10_, Visby, respiratory health effects, asthma, emergency department visits, road dust, limestone

## Abstract

The old Swedish city Visby, located on the island Gotland, has, for several years, reported higher PM_10_ concentrations than any other city in Sweden. In Visby, local limestone is used, both in road paving and as sand used for anti-slip measures, resulting in a clear annual pattern of PM_10_ with the highest concentrations during winter/spring when studded tires are allowed. This study analyzes the short-term associations between PM_10_ and daily number of patients with acute respiratory problems (ICD–10 diagnoses: J00–J99) seeking care at the hospital or primary healthcare units in Visby during the period of 2013–2019. The daily mean of PM_10_ was on average 45 µg m^−3^ during winter/spring and 18 µg m^−3^ during summer/autumn. Four outcome categories were analyzed using quasi-Poisson regression models, stratifying for period and adjusting for calendar variables and weather. An increase in respiratory visits was associated with increasing concentrations in PM_10_ during the summer/autumn period, most prominent among children, where asthma visits increased by 5% (95% CI: 2–9%) per 10 µg m^−3^ increase in PM_10_. For the winter/spring period, no significant effects were observed, except for the diagnose group ‘upper airways’ in adults, where respiratory visits increased by 1% (95% CI: 0.1–1.9%) per 10 µg m^−3^ increase. According to the results, limestone in particles seem to be relatively harmless at the exposure concentrations observed in Visby, and this is in line with the results from a few experimental and occupational studies.

## 1. Introduction

The old Swedish city Visby, located on the island Gotland, has, for several years, reported higher PM_10_ concentrations than any other city in Sweden. In Visby, local limestone is used, both in road paving and as sand used for anti-slip measures, resulting in a clear annual pattern of PM_10_ with the highest concentrations during winter/spring when studded tires are allowed. The potential health effects of this road dust have been discussed in local news media and by the authorities, which is why this study was initiated.

Particulate matter (PM_10_), defined as particles with an aerodynamic diameter smaller than, or equal to, 10 µm, cause a lot of detrimental health effects when inhaled. Meta-analyses have shown that short-term exposure to PM_10_ is linked to increased mortality [1,2]. Short-term respiratory effects in terms of increased number of hospital admissions and emergency department visits for pneumonia associated with increased concentrations of PM_10_ have also been shown in a literature review [3]. Additionally, a systemic review of time-series studies focusing on air pollution-induced hospital admissions and emergency room visits for asthma indicates an increased risk associated with an increased concentration in PM_10_ [4]. Associations between increased concentrations of PM_10_ and hospital admissions for asthma and exacerbations of chronic obstructive pulmonary disease have also been shown in London during the period of 2008–2010 [5]. 

PM_10_ constitutes a mixture of both natural and anthropogenic origins, both locally generated and long-distance transported. The chemical composition and size distribution of PM_10_ vary greatly depending on the origin and the formation processes in the atmosphere. Chemical analyses of the components constituting PM_10_ indicate a large variety of different substances, including elemental carbon (EC), organic carbon (OC), water soluble compounds, such as sulfate (SO_4_^2−^), chloride (Cl^−^), nitrate (NO_3_^−^), ammonium (NH_4_^+^), and also minerals and metals [6,7,8,9].

The health effects caused by exposure to PM_10_ are highly dependent on the chemical composition of the particles. Studies analyzing the health effects associated with specific chemical components point out some particularly detrimental substances. In both epidemiological and toxicological studies, combustion-related particles have been suggested to cause particularly serious health effects [10,11,12,13,14,15,16]. In a meta-analysis, focusing on associations between short-term exposure to PM_2.5_ (particles smaller than, or equal to, 2.5 µm), its constituents and mortality, using city-specific estimates in [17], the factors that could explain the heterogeneity were analyzed. The difference in chemical composition of PM_2.5_ was considered to be an important factor regarding differences in effect estimates. Significant associations between short-term exposure and mortality were found for several constituents of PM_2.5_, and the strongest and most consistent associations were found for elemental carbon (indicator of traffic emissions) and potassium (indicator of wood combustion) [17]. In another meta-analysis, the health effects associated with exposure to different constituents of PM_2.5_ were analyzed from both a short- and a long-term perspective. Effects on mortality and morbidity were analyzed in terms of “all natural”, “cardiovascular”, and “respiratory” outcomes. Several statistically significant associations between specific components in PM_2.5_ and different health endpoints were found. Among these components, black carbon and organic carbon were most consistently and robustly associated with both “all natural” and “cardiovascular” mortality and morbidity. Other components with potentially harmful effects on the cardiovascular system included nitrate, sulfate, zinc, silicon, iron, nickel, vanadium, and potassium. Harmful effects on the respiratory system were also found for nitrate, sulfate, and vanadium [18]. 

The health effects associated with the coarse fraction of PM_10_ (PM_2.5–10_) have been analyzed in a number of studies. In a review study [19], based on several studies analyzing the health effects associated with exposure to particles in the coarse fraction, the short-term effects of PM_2.5–10_ on chronic obstructive pulmonary disease, asthma, and respiratory admissions were stronger than, or as strong as, the corresponding effects associated with exposure to particles in the fine fraction. There was also support for an association between exposure to PM_2.5–10_ and cardiovascular admissions. However, large variations in the effect estimates were shown for the studies that were analyzed. Consequently, it is likely that the chemical composition of PM_2.5–10_ is crucial for the toxicological effects, and for coarse PM, it may vary a lot from place to place and also between seasons [19]. In another review study focusing on health effects caused by exposure to road dust particles, a total of 46 studies from the U.S., Europe, Iran, China, Hong Kong, Korea, and Japan were selected for further analysis. These studies differed in terms of study design, but, overall, it turned out that road dust particles had particularly harmful effects on the respiratory system. The most common chemical components referenced in these studies were lead, platinum-group elements (platinum, rhodium, and bohrium), aluminum, zinc, vanadium, and polycyclic aromatic hydrocarbons [20]. 

The short-term health effects associated with coarse PM in Stockholm have been addressed in a few studies. In a study from Stockholm, based on short-term mortality effects of exposure to PM_2.5–10_ during the period of 2000–2008, a statistically significant excess risk was shown in a single-pollutant model during November–May, while the excess risk during June–October was smaller and non-significant [21]. Statistically significant excess risks in mortality associated with short-term exposure to PM_2.5–10_ has also been shown in another study from Stockholm, based on the period from 2000 to 2016. Except from being significant in a single-pollutant model, a statistically significant excess risk was also shown in a multi-pollutant model adjusting for ozone (O_3_) and nitrogen dioxide (NO_2_) [22]. When the observations were stratified into different seasons, it turned out that the greatest risk increase associated with PM_2.5–10_ occurred during the spring (March–May) [23]. The content of PM_10_ in Stockholm has a clear seasonal pattern with the highest concentrations measured during springtime, and where particles originating from road abrasion constitute up to 90% of the local contribution of PM_10_ [24]. The higher increase in risk in Stockholm during springtime [21,23], indicated a particularly higher toxicity associated with road dust particles, in comparison with PM_2.5–10_ of other origins. In addition, short-term increases in the concentration of PM_10_ has also been associated with the daily number of emergency room visits for asthma in Stockholm [25]. 

In this present study, short-term health effects associated with exposure to PM_10_ in Visby, Sweden, have been analyzed. Visby is located on the island of Gotland in the Baltic Sea east of the southern part of the east coast of the Swedish mainland. According to Swedish standards, relatively high concentrations of PM_10_ have been measured in Visby for several years. The PM_10_ concentrations exhibit a clear annual pattern with the highest concentrations during winter/spring, and with the very highest concentrations during March. In Gotland, limestone is used, both in road paving and as sand used for anti-slip measures, and this annual pattern in PM_10_ concentrations is largely caused by limestone particles which are formed by road abrasion, especially when using studded tires. In Visby, there are also some cobblestone streets, and this type of pavement has been associated with much higher road dust emissions than asphalt-paved roads [26]. The concentrations in PM_10_ are considerably lower during summer and autumn, and those particles are to a lesser extent locally generated. The health effects associated with exposure to PM_10_ largely consisting of limestone particles have not been analyzed to any great extent. However, the health effects associated with mineral particles from desert dust were analyzed in a number of cities in southern Europe during the period of 2001–2010 [27]. Short-term associations with mortality and hospital admissions in connection with desert dust outbreaks were calculated by estimating the PM_10_ concentrations originating from desert dust. Statistically significant excess risks associated with PM_10_ from desert dust were found for natural and cardiovascular mortality, and for respiratory hospital admissions in the age group 0–14 years [27]. Statistically significant excess risks of natural mortality and hospital admissions associated with PM_10_ from Saharan dust were also shown in Sicily based on calculations in the four largest cities and three macro areas [28]. The particles originating from desert dust can consist of very different chemical compositions depending on mineral formation processes and the presence of specific minerals [29], and health effects associated with exposure are strongly dependent on composition parameters [30]. 

During springtime (February–April) in Visby, the percentage calcium content in PM_10_ has been shown to constitute between 15 and 40% of the total particulate mass. In in vitro tests, where cell cultures were exposed to particles containing either calcium carbonate (CaCO_3_) or silicon dioxide (SiO_2_), the effects in terms of decreased cell viability and inflammatory response were considerably lower for CaCO_3_ in comparison with SiO_2_ [31]. Additionally, in a study from Indonesia, where the relationship between limestone dust exposure and lung function capacity among workers in the limestone processing industry was analyzed, there was no association observed [32]. In experimental studies, particle samples consisting primarily of quartz have been among the most potent in inducing cytotoxicity and pro-inflammatory responses in human bronchial epithelial cells and macrophages, whereas the differences in potency are less well established for other minerals [33]. 

The chemical composition of PM_10_ in Visby, with a large proportion of CaCO_3_, especially during winter and spring, causes the population to be exposed to particles with a rather unique chemical composition [34]. The purpose of this study was to analyze the short-term associations between PM_10_ and respiratory emergency department visits in Visby. These analyses were divided into two time periods: winter/spring (January–April) and summer/autumn (May–December). The reason for this division was to determine the differences in effect estimates depending on the percentage content of CaCO_3_ in PM_10_, which was the highest during winter/spring. 

## 2. Materials and Methods

This study analyzes the short-term associations between PM_10_ daily means during 2013–2019 and the daily number of patients with acute respiratory problems (ICD–10 diagnoses: J00–J99) seeking care at the hospital (emergency department or specialist clinic) or a primary healthcare center (including one on-call unit). Data were collected from Gotland Healthcare Administration in Visby for the different care facilities in the area. The collected data also contained date of visit, patient’s age, healthcare unit, and diagnosis, and included only the population registered in Gotland. The diagnose codes were grouped into upper and lower respiratory tract diseases (J0, J3 and J1, J4, respectively), and asthma (ICD–10: J45, J46). The diagnose data were also separated into the two age groups of 0–17 years and >17 years. In Table 1, the numbers of respiratory diagnoses, grouped into the above-mentioned diagnose codes, are presented for different health care units in Visby from 2013 to 2019. Note that “all respiratory” (ICD-10: J00–J99) refers to a total of ten categories, and it is not limited to “upper airways”, “lower airways”, and “asthma”. Therefore, “all respiratory” does not correspond to the sum of these three categories. Additionally, asthma also falls into the category “lower airways”, and consequently, “all respiratory” can take values both greater and less than the sum of “upper airways”, “lower airways”, and “asthma”.

**Figure 1:**
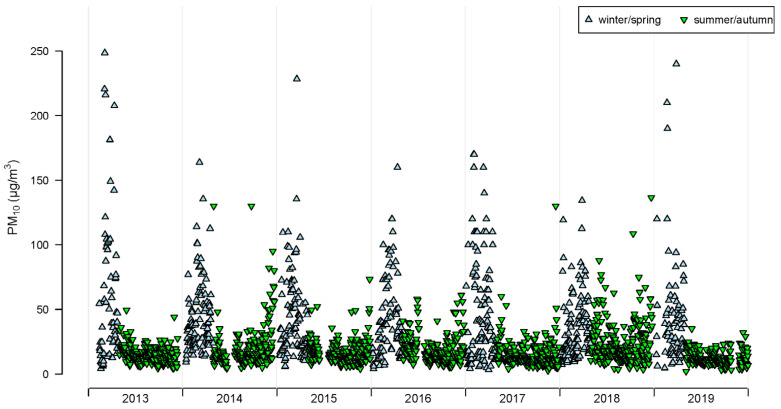
Daily mean values of PM_10_ (µg m^−3^) in Visby during the period of 2013–2019.

Data regarding the daily concentrations of PM_10_ were collected from Region Gotland and covered the years 2013–2019. For the same time period, data on temperature and relative humidity were collected from the Swedish Meteorological and Hydrological Institute (SMHI). The PM_10_ concentrations were measured in a central part of Visby by Region Gotland using a gravimetric measurement method called TEOM 1400AB (Tapered Element Oscillating Microbalance), and daily averages of temperature and relative humidity were collected from SMHI’s weather station located at Visby Airport. Summary statistics on meteorological variables and PM_10_ concentrations are presented in Table 2, and PM_10_ concentrations are also illustrated in Figure 1. The data that have been used are available at the Swedish Meteorological and Hydrological Institute (SMHI) [35]. 

Short-term associations between daily numbers of registered visits regarding respiratory tract diseases and fluctuating concentrations in PM_10_ were analyzed using quasi-Poisson regression models. The regression models adjusted for long-term trends using a penalized spline function with 4 degrees of freedom (d.f.) per year, and penalized splines (4 d.f.) were also used to control for temperature and relative humidity, thus allowing for non-linear associations. The models also adjusted for a varying frequency of visits with respect to weekday patterns and national holidays (factor variables). The concentrations in PM_10_ were analyzed with a two-day running mean value representing the day of the visits and the previous day (lag01), which were also used for temperature and relative humidity. The effects of PM_10_ on the daily number of respiratory diagnoses at the healthcare units in Visby were estimated assuming a linear association, but possible non-linear effects were also investigated by applying a penalized spline function (4 d.f.) for PM_10_. An interaction variable representing winter/spring and summer/autumn periods was used to separate the effects of PM_10_ during these periods, which were defined as January–April and May–December, respectively.

All analyses were performed in the programming language R (version 4.1.2, The R Foundation for Statistical Computing, Vienna, Austria) with the package mgcv (for penalizing splines).

## 3. Results

**Figure 2:**
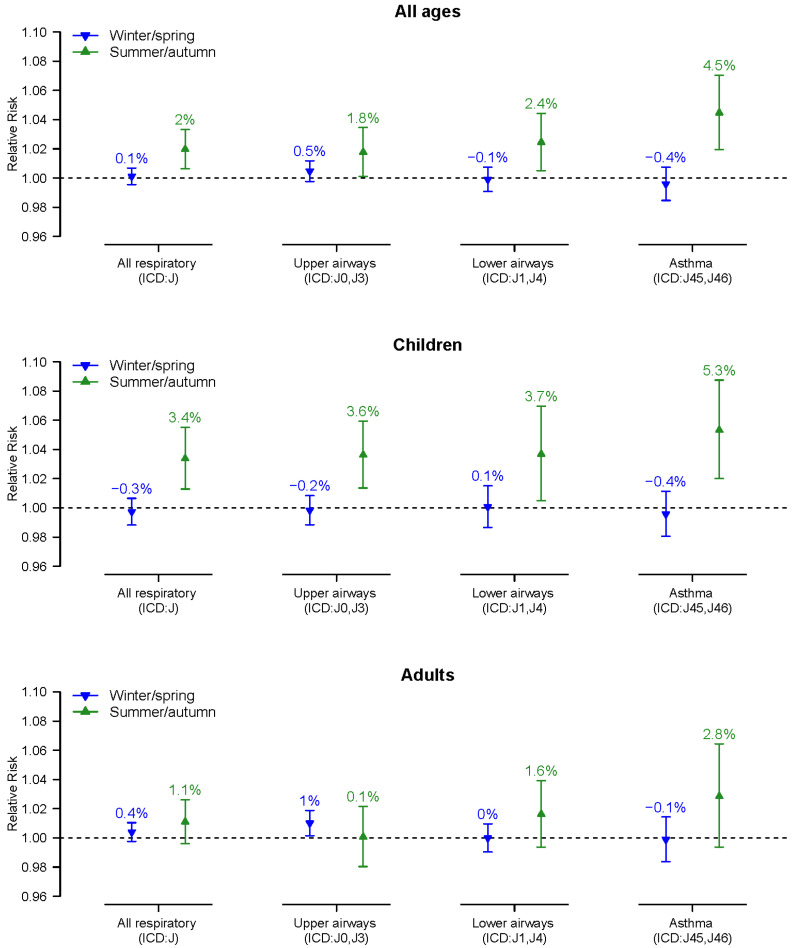
The relative increase (RR with 95% CI) in respiratory visits at all healthcare units in Visby associated with a 10 µg m^−3^ increase in PM_10_ during the period of 2013–2019. All ages at the top, children (0–17 years) in the middle, and adults (>17 years) at the bottom. These analyses are divided into all respiratory diseases (ICD-10: J), upper airways (ICD-10: J0, J3), lower airways (ICD-10: J1, J4), and asthma (ICD-10: J45, J46) during winter/spring (January–April) and summer/autumn (May–December).

The results from the regression models are presented in Table 3 and Figure 2. They show that an increase in all visits regarding respiratory diseases was associated with increasing concentrations in PM_10_ during the defined summer/autumn period. The effects during summer/autumn were most prominent among children and for visits diagnosed with asthma, but significant effects for children were observed in all studied disease groups. Statistically significant effects during the winter/spring period were, however, not seen except for the diagnose group ‘upper airways’ in adults. Figure 2 illustrates the estimated relative increase (with 95% confidence intervals) of daily number of visits for respiratory diseases associated with a 10 µg m^−3^ increase in PM_10_ for the studied age and diagnose groups registered at all healthcare facilities in Visby divided into all ages, children (0–17 years), and adults (>17 years). These analyses are divided into all respiratory diagnoses, upper airways, lower airways, and asthma, which, in turn, are divided into winter/spring (January–April) and summer/autumn (May–December). All estimated relative risks with corresponding 95% confidence intervals are tabulated in Table 3.

**Figure 3:**
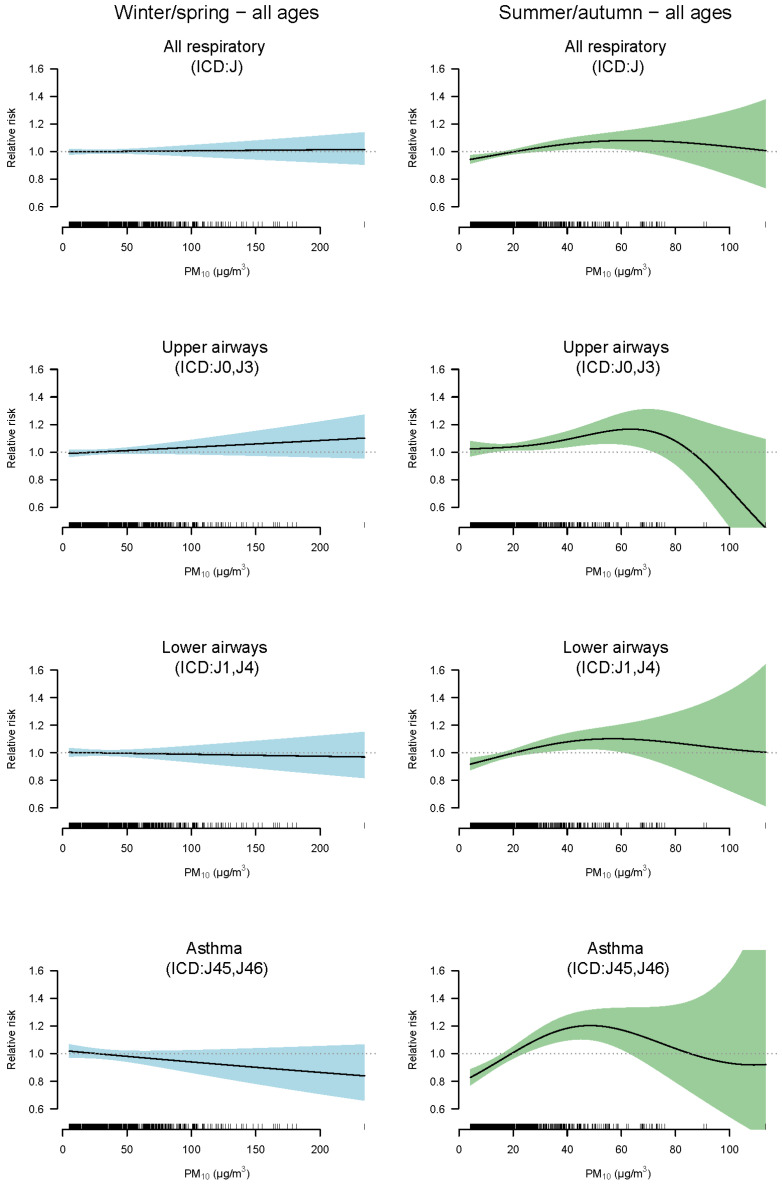
The relative increase (RR with 95% CI) in respiratory visits (all ages) in Visby with concentrations in PM_10_ (lag 01) estimated with regression models allowing for non-linear associations. These analyses are divided into “all respiratory diseases”, “upper airways”, “lower airways”, and “asthma” during winter/spring (January–April) and summer/autumn (May–December).

In Figure 3, the relative increase in respiratory visits in Visby are illustrated, allowing for non-linear associations with the concentrations in PM_10_ (all ages). For the summer period, the models reveal associations with an elevated risk of visits with increased concentrations in PM_10_, but the effects attenuate or become negative with the highest concentrations. For asthma diagnoses and lower airways, there were positive slopes up to the 97th and 98th percentile (48–57 µg m^−3^) of PM_10_ concentrations (lag01). For upper airways and all respiratory diagnoses, the slopes became negative above 62 µg m^−3^ (99th percentile for PM_10_ lag01 during the summer/autumn period). Consequently, the few high PM_10_ outliers (Figure 1) observed during the summer/autumn period were not associated with a high relative risk (Figure 3). For the winter/spring period, no non-linear associations were recognized, nor any significant effects, similar to that revealed by the linear models (all ages).

## 4. Discussion

### 4.1. Differences in Relative Risks during Winter/Spring and Summer/Autumn

This study was initiated by the fact that Visby has one of the highest concentrations of PM_10_ in Sweden and has exceeded the current environmental quality standard. However, the concentrations in PM_10_ showed large seasonal variations with an average concentration during winter/spring that was 2.5 times greater than during the rest of the year (summer/autumn). The relative risks of respiratory diseases associated with a 10 µg m^−3^ increase in PM_10_ during winter/spring showed a positive statistically significant relationship in only one regression model. Contrariwise, the corresponding relative risks during summer/autumn showed statistically significant relationships in all estimates when including all ages and when including children only. The difference in the chemical composition of PM_10_ during the year is, therefore, of crucial importance in terms of differences in health effects throughout the year.

The relative risks observed for a 10 µg m^−3^ increase in PM_10_ (lag01) during summer/autumn are high in comparison with the meta estimate of 1% per 10 µg m^−3^ increase reported for emergency room visits and hospital admissions associated with asthma-like problems [4].

During the winter/spring period, with a large proportion of road dust, the proportion of combustion-related components and endotoxins will be much lower than during the summer/autumn period. Combustion-related components (such as EC, OC and nitrates) [36] and endotoxins [37] are two types of pollutants associated with respiratory hospital admissions and asthma exacerbations. In vitro studies have also shown that allergic responses were more associated with the organic (PAH) fraction of particles, whereas inflammatory responses were more associated with endotoxins and metals [38]. However, for PM_10_ in Visby, actual analyses of these components are lacking.

### 4.2. The Formation Processes and Chemical Compositions of PM_10_ in Visby

PM_10_ in Visby consists of a mixture of different sources that vary throughout the year. The usual particle sources include secondary long-distance transported organic and inorganic aerosols, sea spray, locally produced particles from combustion, road dust, agriculture, plants, and construction sites. The high concentrations of PM_10_ in Visby during winter and spring are, however, caused by particles generated from road dust. In Visby, limestone has been mixed into the asphalt in the municipal road network, and crushed limestone and natural sand with a high content of limestone are used as anti-slip agents on roads, cycle paths, and parking spaces. Limestone is a sedimentary rock with low resistance, which leads to greater particle generation compared with more durable rocks. Moreover, in the inner city of Visby, there are several cobblestone streets. Higher particle emissions from cobblestone streets compared to asphalt-paved streets have been shown, and it is probably due to a combination of the roughness of the cobblestones and the filling material between the stones [26]. However, the few high values in PM_10_ observed outside the defined winter period (Figure 1) may also represent wind-blown mineral dust or sea salt. These particles represent a smaller proportion during the road dust period during winter/spring and a larger proportion during summer/autumn.

### 4.3. Policy Implications

From a policy point of view, selecting the most appropriate action strategies to reduce PM_10_ and its health effects in Visby should focus on its chemical components and not solely on the total concentrations. Limestone particles seem to be relatively harmless at studied exposure concentrations according to the results in this study and previous studies [31,32]. Taking measures specifically aimed at reducing emissions of limestone particles is probably not effective from a health perspective. 

With regard to the choice of action strategies to reduce the concentrations of PM_10_ in Visby, actions should be focused on what can provide the greatest possible health benefits. However, factors in terms of cost-effectiveness and the ability to implement certain measures also need to be considered. The reduction measures should be aimed at reducing road dust particles in general, instead of measures specifically aimed at reducing limestone particles. Road dust contains a combination of materials originating from mechanical wear of road surfaces (asphalt or cobblestones), tires, and brakes. Measures aimed at reducing road dust particles include reduced use of studded tires, reduced speed, dust binding, and vacuum cleaning.

### 4.4. Strengths and Limitations of This Study

A strength of this study is that the diagnose data should reflect short-term variations in respiratory problems in a good way since access to health care is good and the cost is moderate for the patient. Moreover, no visits by tourists were included in the analyses, which is important since Visby is a World heritage city with many visitors. This study is also special in the sense that the population every year is exposed to two periods with very different chemical composition of PM_10_. 

A limitation of this study is that PM_10_ is the only air pollutant that was measured over the study period, and no multi-pollutant models with other air pollutants have been possible to produce. Consequently, it is not possible to exclude confounding effects from other pollutants e.g., ozone. Additionally, confounding effects from pollen are also possible, especially during spring and summer. Another limitation is the use of only one centrally located measuring station in Visby for the measurements of the concentrations in PM_10_, and only one measuring station located at Visby Airport for the measurements of the weather variables. With only one measuring station, the geographical variation in concentrations and meteorology cannot be captured. Even if most of the study population live in the city, the random misclassification of the short-term variation in concentrations could dilute the observed associations between exposure and relative risk. 

## 5. Conclusions

According to Swedish standards, relatively high concentrations in PM_10_ have been measured in Visby for several years. The PM_10_ concentrations exhibit a clear annual pattern with the highest concentrations during winter/spring, and with the very highest concentrations during March. Since limestone is used in road paving and as sand used for anti-slip measures, this annual pattern is mainly caused by limestone particles which are formed by road abrasion, especially when using studded tires. 

During summer/autumn, the short-term health effects associated with increasing concentrations in PM_10_ were very clear with statistically significant associations in most cases. During winter/spring, however, the corresponding health effects were in most cases not statistically significant. An overall conclusion of this study is that limestone in particles, which mainly contribute to the very high concentrations of PM_10_ during wintertime, seem to be relatively harmless at the exposure concentrations observed in Visby. 

## Figures and Tables

**Table 1:** The numbers of respiratory diagnoses at different healthcare units in Visby during the period of 2013–2019.

Healthcare Unit	All Respiratory (ICD-10: J00–J99)	Upper Airways (ICD-10: J0, J3)	Lower Airways (ICD-10: J1, J4)	Asthma (ICD-10: J45, J46)
Visby hospital (acute care visits at specialist clinics)	37,736	14,751	16,636	9297
Primary healthcare on-call unit and hospital emergency department (ERD)	10,886	5347	3390	1076
Primary healthcare centers	29,389	19,420	7888	3090

**Table 2:** Summary statistics (including standard deviation (SD) and number of observations (N)) of PM_10_ (µg m^−3^), temperature (°C), and relative humidity (%) in Visby during the period of 2013–2019.

	Min.	25th Percentile	Median	Mean	75th Percentile	Max.	SD	N
PM_10_ (whole year) (µg m^−3^)	2.2	11.8	17	26.1	29	248.6	26.1	2140
PM_10_ (winter/spring) (µg m^−3^)	3.5	18.3	34	45	59.1	248.6	37.5	657
PM_10_ (summer/autumn) (µg m^−3^)	2.2	10.5	14.8	18	21	136.6	13	1483
Temperature (whole year) (°C)	−10.4	2.9	7.2	8.2	14.2	25.8	6.8	2702
Relative humidity (whole year) (%)	46.7	76.7	85.2	83.9	92.6	100	10.7	2674

**Table 3:** Relative risk of a 10 µg m^−3^ increase in PM_10_ for the studied age and diagnose groups registered at all healthcare facilities in Visby divided into all ages, children (0–17 years), and adults (>17 years).

Diagnose Group	Age Group	Relative Risk [95% CI] Winter/Spring	Relative Risk [95% CI] Summer/Autumn
All respiratory (ICD-10: J)	All ages	1.001 [0.996–1.007]	1.020 [1.006–1.033]
All respiratory (ICD-10: J)	Children (0–17 yr)	0.997 [0.988–1.007]	1.034 [1.013–1.055]
All respiratory (ICD-10: J)	Adults (>17 yr)	1.004 [0.998–1.010]	1.011 [0.996–1.026]
Upper airways (ICD-10: J0, J3)	All ages	1.005 [0.998–1.012]	1.018 [1.001–1.035]
Upper airways (ICD-10: J0, J3)	Children (0–17 yr)	0.998 [0.988–1.008]	1.036 [1.014–1.059]
Upper airways (ICD-10: J0, J3)	Adults (>17 yr)	1.010 [1.001–1.019]	1.001 [0.980–1.021]
Lower airways (ICD-10: J1, J4)	All ages	0.999 [0.991–1.007]	1.024 [1.005–1.044]
Lower airways (ICD-10: J1, J4)	Children (0–17 yr)	1.001 [0.987–1.015]	1.037 [1.005–1.070]
Lower airways (ICD-10: J1, J4)	Adults (>17 yr)	1.000 [0.990–1.010]	1.016 [0.994–1.039]
Asthma (ICD-10: J45, J46)	All ages	0.996 [0.985–1.007]	1.045 [1.020–1.070]
Asthma (ICD-10: J45, J46)	Children (0–17 yr)	0.996 [0.980–1.011]	1.053 [1.020–1.087]
Asthma (ICD-10: J45, J46)	Adults (>17 yr)	0.999 [0.984–1.014]	1.028 [0.994–1.064]

## Data Availability

Health registry data can be requested from the Gotland Health Care Administration. Air pollution data and meteorological data can be requested from the Swedish Meteorological and Hydrological Institute (SMHI).
